# Effects of southern rice black-streaked dwarf virus on the development and fecundity of its vector, *Sogatella furcifera*

**DOI:** 10.1186/1743-422X-10-145

**Published:** 2013-05-12

**Authors:** Zhi Tu, Bing Ling, Donglin Xu, Maoxin Zhang, Guohui Zhou

**Affiliations:** 1 College of Natural Resources and Environment, South China Agricultural University, Guangzhou 510642, China

**Keywords:** White-backed planthopper, Life parameter, Virus–vector interaction

## Abstract

**Background:**

*Southern rice black-streaked dwarf virus* (SRBSDV) threatens rice production in China and Vietnam. The virus is vectored by the migrating white-backed planthopper (WBPH, *Sogatella furcifera*) in a circulative, propagative, and persistent manner. A persistently-transmitted plant virus might affect its vector’s development and fecundity directly by infecting the vector itself and/or indirectly altering the host plant. This study evaluated the direct and indirect effects of SRBSDV on WBPH performance to better understand the virus–vector–host plant relationship in terms of its effects on the biological parameters of the vector.

**Methods:**

Three experimental WBPH populations were established. Viruliferous and non-viruliferous populations were fed on SRBSDV-infected rice seedlings for 48 h as first-instar nymphs; infection status was confirmed by RT–PCR after they died. The control population was fed on healthy rice. Each insect was individually transferred to a healthy rice plant grown in a glass tube at 20°C, 25°C, or 28°C. Life parameters, including nymphal duration, survival rate, adult sex ratio, macropterous proportion, longevity, and oviposition amounts, of each population were measured at each temperature.

**Results:**

The life parameter data indicated that SRBSDV and infected rice plants adversely influenced WBPH; the effects were temperature dependent. Compared with the control population, viruliferous populations showed significant changes, including prolonged nymphal stages and reduced survival rates at 20°C, while the non-viruliferous population had higher survival rates at 20°C and lower rates at 28°C compared with the control. Both populations had significantly shorter adult life spans at 25°C and lower oviposition amounts at 28°C relative to the control.

**Conclusions:**

Both SRBSDV-infection and feeding on infected rice plants affected vector performance. Although a longer nymphal period benefits viral acquisition and transmission by nymphs and might increase rice infection rate, in general, SRBSDV infection of the vectors and host plants was unfavorable to WBPH population expansion.

## Introduction

*Southern rice black-streaked dwarf virus* (SRBSDV) is a novel species in the genus *Fijivirus*, family *Reoviridae*, proposed by Zhang et al. [1] and Zhou et al. [2] in 2008. The viral particles are icosahedral, double-layered virions with a diameter of approximately 70 nm and contain ten double-stranded RNA (dsRNA) genomic segments named S1 to S10 [3]. This virus, first found in Guangdong Province, China, has spread rapidly throughout southern China and northern Vietnam, and poses a great threat to rice production in these areas.

The white-backed planthopper (WBPH, *Sogatella furcifera*, Delphacidae, Hemiptera), a typical large-scale migratory rice pest in Southeast Asia, is currently the only confirmed vector of SRBSDV. The virus can abundantly propagate in the insect body and is transmitted by WBPH in a persistent manner, but cannot be transmitted through eggs [2]. Pu et al. [4] reported that 83% of the second-generation population of WBPH adults propagated on SRBSDV-infected rice plants became viruliferous. The minimum virus acquisition and inoculation access periods were 5 and 30 min, respectively, for both WBPH nymphs and adults, and the circulative transmission period of the virus in WBPH ranged from 6–14 days [4]. Most viruliferous individuals transmitted the virus with intermittent periods ranging from 2–6 days, and a single viruliferous individual could transmit it to 8–25 rice plants within a 5-day period [4]. WBPH can transmit SRBSDV from rice to maize seedlings, but it rarely acquires the virus from infected maize [5].

A disease cycle of the SRBSDV-caused rice disease was proposed by Guo et al. [6]. Briefly, SRBSDV and its vector, WBPH, overwinter in a warm tropical or sub-tropical area. The viruliferous WBPH adults bring the virus from south to north (in the Northern Hemisphere) through their migration in early spring, transmit it to rice seedlings in the newly colonized area, and lay eggs. The nymphs hatch and feed on the infected seedlings, and a high proportion of the next generation of WBPHs become viruliferous. Their dispersal leads to secondary infections of the virus and severe outbreaks of the viral disease.

SRBSDV can be considered an insect virus because it circulates and propagates within WBPH. Ultra-thin section electron microscopy showed that a large amount of virions were found in the midgut, coelom, and salivary gland of viruliferous WBPHs [7]. Studying the virus’ effects on its vector is crucial to better understanding its epidemiology and to designing control strategies [8,9].

In recent years, increasing attention has been paid to the virus–vector–host plant relationship, especially to the interactions between vector and virus. A virus may have favorable, neutral, or adverse effects on its vector, either directly via infection or indirectly via virus-induced alterations, such as morphological or nutritional changes, in the host plant [10,11]. However, the nature of the interaction between SRBSDV and WBPH is still unclear.

In this study, we established three experimental populations of WBPH: viruliferous and non-viruliferous populations fed on virus-infected rice and a control population fed on healthy rice. The life parameters of the three populations were measured at three temperatures and comparatively analyzed to reveal the influences of SRBSDV and the infected rice on WBPH, gain insight into epidemical mechanism of the virus and provide theoretical support for disease-controlling strategies.

## Materials and methods

### Tested plants, virus, and insects

The rice cultivar Taichung Native 1 (TN-1) was used in this study. Water planting of the rice was conducted according to Yoshida et al. [12]. Briefly, the seeds were germinated and sown in a 2-L beaker filled halfway with culture solution till the two-leaf seedling stage. A number of seedlings in uniform size were selected and individually transferred into culture tubes (2.5 cm in diameter, 25 cm in length). The roots of each seedling were wrapped in a sponge strip and immersed in the culture solution. The seedlings were cultured in a growth chamber at 27 ± 1°C, under relative humidity of 75 ± 5% and photoperiod of 14 h light/10 h dark.

The SRBSDV isolate was collected from infected field rice in Guangzhou, Guangdong Province, China, and confirmed by RT–PCR. The virus was transmitted by WBPH to, and maintained on, rice plants grown in insect-proof greenhouses.

WBPH adults were collected from healthy field plants in Guangzhou and propagated on healthy TN-1 plants grown in insect-proof greenhouses at 27 ± 1°C, under relative humidity of 75 ± 5% and photoperiod of 14 h light/10 h dark. SRBSDV cannot be transmitted via WBPH eggs. To confirm that WBPH were not viruliferous, the offspring (second generation) of the virus-free original insects (confirmed by RT–PCR after they died) were reared on the same plants until the fourth instar. Twenty of them were selected and raised on healthy rice seedlings in culture tubes and then transferred to tillering healthy plants after eclosion. From this population, six pairs of newly-emerged third-generation males and females were placed on a healthy plant to mate and reproduce. Their offspring (the fourth generation) were used in this study.

### Measurement of the life parameters of WBPH

The three largest groups of newly-hatched, fourth-generation nymphs were chosen for the following experiments at 20°C (below the optimal growth temperature, OPT), 25°C (the OPT) and 28°C (above the OPT). For each group, about eighty nymphs were placed on tillering SRBSDV-infected rice plants (inoculated at the three-leaf stage and infection confirmed by RT–PCR) for 48 h. Forty nymphs were fed on healthy plants for 48 h as the control. After feeding, each nymph was individually transferred to a healthy rice seedling at the three-leaf stage in a glass tube in a growth chamber under the conditions described above. Observations were recorded every 12 h to monitor the ecdysis process of the insects until their eclosion to the adult stage. Data included the duration and death rates of each instar. The adult sex ratios and proportion of macropterous individuals were calculated after eclosion. The newly obtained female and male adults were randomly paired and transferred to tillering healthy plants for mating and oviposition. Observations of hatching began 5 d later. Nymph numbers were recorded daily until no new nymphs were observed for three consecutive days. Then, the numbers of unhatched eggs in the stalks were counted, and oviposition and hatching rates were calculated.

Every tested WBPH individual was subjected to RT–PCR after its death, regardless of developmental stage, to detect SRBSDV. Each insect fed on a diseased plant was assigned to either a viruliferous or a non-viruliferous population, based on its RT–PCR result. The WBPH fed on healthy plants were defined as the control population. These three populations were statistically compared. To analyze survival rate, proportion of macropterous insects, and adult sex ratio, each population was equally (or nearly equally) and randomly divided into three groups to allow the comparison of mean percentage values among populations.

### Virus detection by RT–PCR

SRBSDV in rice plants or WBPHs was detected using the duplex RT–PCR method described by Wang et al. [13]. Briefly, total RNA was extracted from 0.1 g of rice leaf tissue or from an individual WBPH and amplified by RT–PCR using two pairs of primers (5′-TTACAACTGGAGAAGCATTAACACG-3′/5′-ATGAGGTATTGCGTAACTGAGCC-3′ and 5′-CGCGTCATCTCAAACTACAG-3′/5′-TTTGTCAGCATCTAAAGCGC-3′) and a One Step RNA PCR kit (AMV) (TaKaRa, Dalian, China) following the manufacturer’s protocol. The sizes of the two expected amplicons were 819 and 682 bp.

### Statistical analysis

Data from the viruliferous, non-viruliferous, and control populations were expressed as means ± standard errors. Percentage data were arcsine square root transformed before being used for an analysis of variance. The Student–Newman–Keuls test (SNK or *q*-test), or the Student’s *t*-test if necessary, was used to detect significant differences at the *P* = 0.05 level between populations with DPS software (ver. 9.5) (Data Processing System, Fujitsu Ltd., China).

## Results

### Effects of SRBSDV and infected rice plants on nymphal duration and survival of WBPH

In the viruliferous population, each nymphal instar and, consequently, the whole nymphal period, lasted longer than in the control at 20°C, but the durations were equal at 25°C and 28°C (Table 1). Compared to the control, the non-viruliferous had significantly longer second instar, third instar, and total nymphal period at 20°C and longer third and fourth instars at 25°C, but not at 28°C. The viruliferous and the non-viruliferous populations had similar total nymphal periods and, in most cases, instar durations at all three temperatures. These results indicate that temperature had a greater effect on nymphal duration when first-instar WBPH were fed on diseased rice for 48 h.

**Table 1:** **Nymphal durations of viruliferous, non-viruliferous, and control populations of *Sogatella furcifera *at different temperatures**

**Temperature**	**Population**	**Number**	Nymphal stage duration (days ± S.E.) *				
			**2nd instar**^**1)**^	**3rd instar**^**2)**^	**4th instar**^**3)**^	**5th instar**^**4)**^	**Total**^**5)**^
20°C	Viruliferous	61	6.9 ± 0.3a	5.3 ± 0.2a	5.0 ± 0.1a	6.4 ± 0.1a	23.6 ± 0.5a
	Non-viruliferous	12	7.8 ± 1.8a	6.3 ± 0.3a	4.8 ± 0.3ab	6.3 ± 0.3ab	25.0 ± 1.5a
	Control	36	3.8 ± 0.1b	3.5 ± 0.1b	4.0 ± 0.2b	5.6 ± 0.2b	16.8 ± 0.4b
25°C	Viruliferous	32	2.4 ± 0.2a	2.2 ± 0.1b	2.5 ± 0.0b	3.6 ± 0.1a	11.0 ± 0.4a
	Non-viruliferous	37	2.4 ± 0.1a	2.6 ± 0.1a	3.6 ± 0.1a	3.6 ± 0.1a	10.6 ± 0.2a
	Control	38	2.4 ± 0.1a	2.1 ± 0.1b	2.4 ± 0.1b	3.4 ± 0.1a	10.2 ± 0.2a
28°C	Viruliferous	41	1.2 ± 0.1a	1.9 ± 0.1a	2.0 ± 0.1a	3.0 ± 0.1a	8.4 ± 0.1a
	Non-viruliferous	36	1.6 ± 0.1a	1.9 ± 0.1a	1.9 ± 0.1a	3.0 ± 0.1a	8.5 ± 0.2a
	Control	38	1.8 ± 0.1a	1.8 ± 0.1a	2.0 ± 0.1a	3.0 ± 0.1a	8.4 ± 0.2a

* In the same column at the same temperature, mean values (± SE) followed by different letters differ significantly at the *P* = 0.05 level by SNK (*q*-test).

^1)^ One way ANOVA: *F* = 45.0, df = 2, *P* = 0.0001 for 20°C; *F* = 0.04, df = 2, *P* = 0.9581 for 25°C; *F* = 2.5, df = 2, *P* = 0.0914 for 28°C.

^2)^ One way ANOVA: *F* = 33.7, df = 2, *P* = 0.0001 for 20°C; *F* = 10.0, df = 2, *P* = 0.0001 for 25°C; *F* = 0.3, df = 2, *P* = 0.7229 for 28°C.

^3)^ One way ANOVA: *F* = 9.0, df = 2, *P* = 0.0003 for 20°C; *F* = 151.9, df = 2, *P* = 0.0001 for 25°C; *F* = 0.5, df = 2, *P* = 0.5825 for 28°C.

^4)^ One way ANOVA: *F* = 5.7, df = 2, *P* = 0.0052 for 20°C; *F* = 3.3, df = 2, *P* = 0.0397 for 25°C; *F* = 0.3, df = 2, *P* = 0.7547 for 28°C.

^5)^ One way ANOVA: *F* = 54.8, df = 2, *P* = 0.0001 for 20°C; *F* = 2.7, df = 2, *P* = 0.0776 for 25°C; *F* = 0.1, df = 2, *P* = 0.8658 for 28°C.

**Figure 1:**
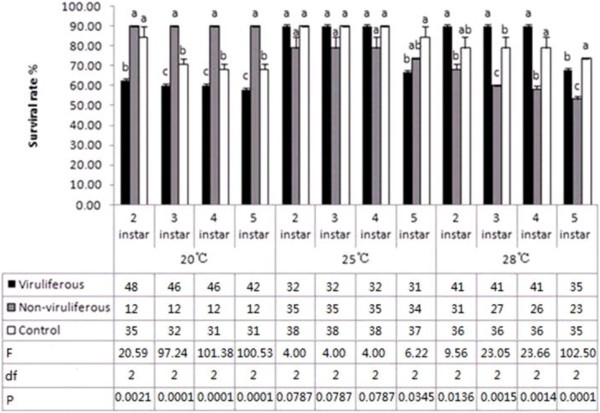
**Survival rates of white-backed planthopper (*Sogatella furcifera*) nymphs at 20°C, 25°C and 28°C.** Sample sizes at 20°C for the viruliferous, non-viruliferous, and control populations were 61, 12, and 36 individuals, respectively; at 25°C were 32, 37, and 38, respectively; and at 28°C were 41, 36, and 38, respectively at 28°C. Values shown are means ± SE. Different letters above the columns for a given instar and temperature indicate that the means that differ significantly at the *P* = 0.05 level by the SNK test. Numbers of surviving nymphs at each instar, as well as the *F*, df, and *P* values, are presented below the columns.

In comparison with the control, the viruliferous population at 20°C had significantly lower survival rates in their nymphal period, whereas the non-viruliferous population had significantly higher survival rates (Figure 1). At 25°C, none of the populations differed significantly in survival rate until the fifth instar, which the viruliferous insects had higher mortality. The viruliferous population at 28°C had a significantly higher survival rate than the control in the third instar and a lower rate in the fifth instar, while the non-viruliferous suffered significantly lower survival rates in instars 3–5. These results indicated that infection of WBPH by SRBSDV negatively influences insect survival at temperatures below the optimum growth temperature (25°C) and late in the nymphal period (instar 5), regardless of temperature. Also, WBPH that ingest infected plants without becoming infected themselves (i.e., the non-viruliferous population) had significantly higher survival rates below the optimal temperature and significantly higher mortality above the optimum growth temperature throughout instars 3–5.

### Effects of SRBSDV and infected rice plants on sex ratio and proportion of macropterous WBPH

**Figure 2:**
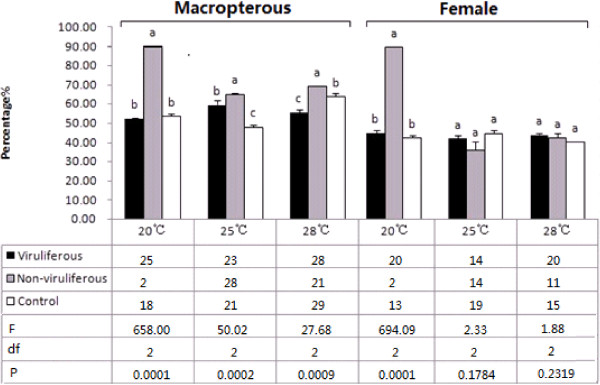
**Proportions of macropterous white-backed planthoppers (*Sogatella furcifera*) and female adults in different populations.** Samples sizes at 20°C for the viruliferous, non-viruliferous, and control populations were 40, 2, and 28 individuals, respectively; at 25°C were 31, 34, and 38, respectively; and at 28°C were 41, 24, and 36, respectively. Values shown are means ± SE. Different letters above the columns for a given temperature indicate means that differ significantly at the *P* = 0.05 level by the SNK test. The numbers of surviving nymphs at each instar, as well as the *F*, df, and *P* values, are presented below the columns.

Compared with the control, the viruliferous population at 20°C had an approximately 1:1 sex ratio and proportion of macropterous adults, while the non-viruliferous population was significantly skewed toward macropterous and female individuals (Figure 2). At 25°C, both the viruliferous and the non-viruliferous populations had significantly more macropterous adults than the control, but neither had significantly altered sex ratios. The viruliferous population at 28°C had fewer and the non-viruliferous one had more macropterous adults than the control, although neither population had significantly changed sex ratios. In general, the infected rice had a greater impact on the non-viruliferous population than on the viruliferous one. The impact was more significant on wing type than on sex ratio and was more prevalent at the lower temperature.

### Effects of SRBSDV and infected rice plants on longevity of WBPH adults

**Figure 3:**
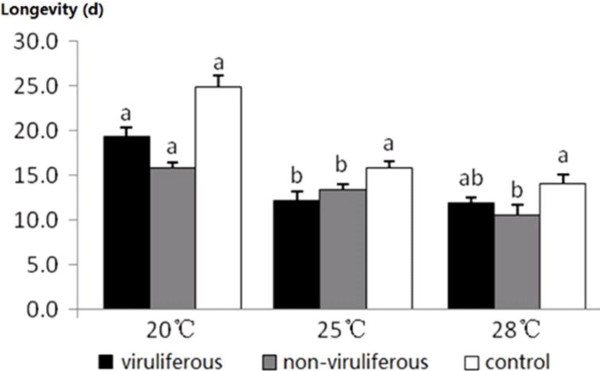
**Longevity of white-backed planthopper (*Sogatella furcifera*) adults at 20°C, 25°C, and 28°C.** Samples sizes at 20°C for the viruliferous, non-viruliferous, and control populations were 40, 2, and 28 individuals, respectively (*F* = 7.2, df = 2, *P* = 0.0018); at 25°C were 31, 34, and 38, respectively (*F* = 5.3, df = 2, *P* = 0.0065); and at 28°C were 41, 24, and 36, respectively (*F* = 3.7, df = 2, *P* = 0.0294). Values shown are means ± SE. Different letters above the columns indicate means that differ significantly at the *P* = 0.05 level by the SNK test.

The longevity of both the viruliferous and the non-viruliferous adults were not significantly altered at 20°C, whereas both were significantly shortened at 25°C, and only the non-viruliferous group had shorter lives at 28°C compared with the control (Figure 3). These results indicated that SRBSDV-infected plants may adversely affect adult longevity of WBPH, and insect viral infection can attenuate the harmful effects of sub-optimal (cold or warm) temperatures on WBPH longevity.

### Effects of SRBSDV and infected rice plants on fecundity of WBPH adults

No significant differences from the control in egg stage duration or hatching rate were found in the viruliferous or non-viruliferous populations, except that the viruliferous group at 28°C had a significantly lower hatching rate (25.4% less) (Table 2). However, oviposition amounts were dramatically reduced at 20°C (41.2% lower for the viruliferous population) and 28°C (viruliferous: 56.1% lower; non-viruliferous: 63.6% lower). At 25°C, the viruliferous population laid 79.7% fewer eggs, but the non-viruliferous population laid nearly the same amount as the control. Similar results were observed in the oviposition rate (egg amount divided by adult longevity). These results suggest that SRBSDV and/or infected plants affected the quantity much more than the quality of WBPH eggs, and the influence varied with temperature. Interestingly, only SRBSDV infection and not the diseased plants themselves affected oviposition of WBPH at its optimum growth temperature (25°C).

**Table 2:** **Fecundity of the white-backed planthopper (*Sogatella furcifera*) adults from viruliferous, non-viruliferous, and control populations**^1)^

**Temperature**	**Population**	**Number**	**Egg amount**^**2)**^	**Oviposition rate (d**^**–1**^**)**^**3)**^	**Egg duration**^**4) **^**(days ± S.E.)**	**Hatching rate**^**5) **^**(%)**
20°C	Viruliferous	12	100.3 ± 14.6*	5.5 ± 0.6*	14.7 ± 0.6	95.6 ± 1.5
	Non-viruliferous	0	-	-	-	-
	Control	11	170.7 ± 17.0	8.7 ± 1.4	14.7 ± 0.2	94.9 ± 2.6
25°C	Viruliferous	6	17.2 ± 7.7b	5.3 ± 1.0b	7.1 ± 0.3a	100 ± 0.0a
	Non-viruliferous	9	83.7 ± 18.5a	10.5 ± 2.0a	8.2 ± 0.4a	96.4 ± 1.6a
	Control	10	84.7 ± 18.1a	8.2 ± 1.5a	8.4 ± 0.3a	96.1 ± 2.1a
28°C	Viruliferous	5	111.6 ± 29.0b	8.4 ± 2.8b	8.6 ± 0.2a	72.5 ± 12.2b
	Non-viruliferous	5	92.6 ± 50.0b	8.7 ± 3.9b	10.4 ± 0.9a	100 ± 0.0a
	Control	12	254.3 ± 39.6a	19.8 ± 2.8a	8.8 ± 0.4a	97.2 ± 1.3a

* indicates a significant difference at *P* = 0.05 level by Student’s *t*-test.

^1)^ Mean values (± SE) in the same column at the same temperature that are followed by different letters differ significantly at the *P* = 0.05 level by the SNK test.

^2)^ One way ANOVA: *F* = 4.0, df = 2, *P* = 0.032 for 25°C; *F* = 4.4, df = 2, *P* = 0.0266 for 28°C.

^3)^ One way ANOVA: *F* = 2.4, df = 2, *P* = 0.114 for 25°C; *F* = 4.4, df = 2, *P* = 0.0304 for 28°C. Oviposition rate equals to the egg amount divided by adult longevity (eggs/day).

^4)^ One way ANOVA: *F* = 2.6, df = 2, *P* = 0.0974 for 25°C; *F* = 3.2, df = 2, *P* = 0.063 for 28°C.

^5)^ One way ANOVA: *F* = 1.8, df = 2, *P* = 0.1958 for 25°C; *F* = 11.4, df = 2, *P* = 0.0008 for 28°C.

## Discussion

As a newly emerged pathogen, SRBSDV poses a severe threat to rice production in China and Vietnam and is currently the only WBPH-transmitted rice virus. In this study, we evaluated the effects of SRBSDV on the life parameters of WBPH and found that both SRBSDV and virus-infected rice plants significantly affected the insect’s performance. Some of the effects were temperature-dependent. These results give insight into the virus–vector–host plant relationship and may help in monitoring, forecasting, and controlling this viral disease.

Many studies have demonstrated that virus-infected host plants can affect the life parameters of insect vectors in different ways [14-17]. For instance, aphids fed on hosts infected with *Potato leaf roll virus* benefited from improved survival rate, fecundity, lifespan, and intrinsic rate of increase compared to those on healthy plants [14]. A population of the invasive tobacco whitefly, *Bemisiatabaci*, fed for 56 d on tobacco plants infected with *Tomato yellow leaf curl China virus* (TYLCCNV) or *Tobacco curly shoot virus* (TbCSV) increased faster in size, had 11–17 times higher fecundity, and a 5–6 times longer lifespan [15]. Conversely, the same insect fed on *Cotton leaf curl virus*-infected cotton leaves lived shorter lives and laid fewer eggs [16,17]. However, these previous studies did not analyze the causal factors of the changes in vector performance: the direct impact from insect viral infection and the indirect impact from virus-induced compositional changes in the diseased plants.

Notably, in our study, not all the WBPH individuals became viruliferous after feeding on infected hosts. Therefore, the insects that had been fed diseased plants were divided into two populations, viruliferous and non-viruliferous, to comparatively investigate the viral effects on the life parameters of WBPH, enabling us to differentiate the direct and indirect virus-on-vector impacts. Moreover, to minimize the experimental error due to genetic variation within the WBPH population being tested, we propagated six pairs of field collected insects to the fourth generation and then used the offspring for life parameter tests.

Unexpectedly, a very short period (2 d) of feeding on infected plants early in the WBPH life cycle (first instar nymph) could significantly affect some life parameters of both the viruliferous and the non-viruliferous populations, including a prolonged nymphal stage duration at 20°C and shortened adult lifespan at 25°C (Table 1, Figure 3). In addition to insect viral infection (a direct effect), the sap components (nutritional substances, toxins, miRNAs, etc.) in the infected host plant (an indirect effect) might alter the growth and development of WBPH. Previous studies have found that viral infection can alter the nutrient content in the plant sap and subsequently change the life parameters and fecundity of vectors feeding on those plants [18,19]. However, because nutrition directly affects insect growth and development, a brief change in the nutrient content is unlikely to inflict such considerable and long-lasting effects, as found in this study. The virus–vector interaction can be very complex, involving insect genetics, insect physiology, and/or viral replication. The non-viruliferous insects may have undergone a transient viral infection that was eliminated during the nymphal period but that actually affected the insects.

SRBSDV can propagate well and circulate in the body of WBPH [20]. When the virions enter the insect’s intestinal tract, they may be digested by intestinal enzymes, releasing genomic dsRNAs that can degrade into small RNA fragments that might trigger RNA interference (RNAi). Some studies have demonstrated the strong RNAi-inducing activities of dsRNAs ingested or injected into the insect’s body [21-24], and a recent report showed that ingested plant miRNAs could regulate gene expression in mammals as well [25]. We found that SRBSDV infection remarkably altered the expression profile of rice miRNAs (unpublished data). Xu et al. [26] confirmed a significant change in the WBPH transcriptome after SRBSDV infection. In this study, the survival rates of nymphs (Figure 1) and wing dimorphisms (Figure 2) were apparently affected by SRBSDV, probably because of damage in functionally-relevant tissues and organs and/or the altered expression of some related genes. More studies will be needed to determine whether SRBSDV and rice miRNAs ingested by WBPH induce RNAi in the insect and consequently affect its life parameters.

The virus-on-vector effects may differ among virus–vector–host combinations. Most insect vectors benefit from feeding on virus-infected host plants; in particular, many non-circulative propagative viruses raise the intrinsic rate of increase or the proportion of macropterous adults in natural population of their vectors [27-30]. For example, the mutualistic symbiosis of *B. tabaci* and TYLCCNV or TbCSV can facilitate whitefly invasion and enhance the viral disease epidemic [15]. In contrast, some circulative propagative viruses adversely affect their vectors by shortening their lifespans or raising their mortality rates [31,32]. In this study, a 2-d feeding on diseased plants significantly lengthened nymphal durations at 20°C (Table 1), changed survival rates at 20° and 28°C (Figure 1), shortened the longevity of viruliferous adults at 25°C and non-viruliferous adult at 25°C and 28°C (Figure 3), and decreased oviposition amounts (Table 2) in WBPH. Although a longer nymphal period benefits viral acquisition and transmission by the nymphs and may increase the plant infection rate, in general, SRBSDV and infected plants are unfavorable for the expansion of the WBPH population. This finding might explain the rapid decrease in SRBSDV occurrence in China in 2011 and 2012 immediately after a period of high prevalence in 2009 and 2010. Similar interactions might also exist between other rice viruses and their vectors and, therefore, lead to intermittent disease epidemics [33]. Further investigation is needed to determine whether the SRBSDV–WBPH relationship restrains viral epidemics and vector populations.

Environmental temperatures can substantially affect insect development and survival. For example, we found faster nymphal maturation in all three WBPH populations at the highest temperature in this study (Table 1). Temperature may also influence virus propagation and the immunological responses in the vector, so virus-induced effects on the vector may vary under different thermal conditions [34]. The differences in the nymphal period and adult lifespan of the viruliferous population (Table 1, Figure 3) suggested that temperature might affect the immunity of WBPH and the propagation of SRBSDV [35]. The differences in nymphal death rate, wing type proportion, and sex ratio between the viruliferous and non-viruliferous populations (Figures 1 and 2) were also temperature-dependent, and whether these differences are associated with RNAi induced by dsRNAs and/or miRNAs in infected rice plants is worthy of future studies.

## Conclusions

To analyze the direct and indirect impacts of SRBSDV on WBPH, a persistent vector of the virus, we divided first-instar insects into viruliferous and the non-viruliferous populations after a 48-h feeding period on virus-infected rice using RT–PCR detection. Both the virus and infected rice plants adversely influenced the vector’s life parameters, and the effects were temperature dependent. These results will be useful to understand the virus–vector–host plant relationship and will be beneficial for monitoring, forecasting, and controlling this viral disease.

## Abbreviations

SRBSDV: Southern rice black-streaked dwarf virus; WBPH: White-backed planthopper; RT–PCR: Reverse transcription–polymerase chain reaction.

## Competing interests

The authors declare that they have no competing interests.

## Authors’ contributions

ZT is a PhD student who carried out the experiments and drafted the manuscript. BL was involved in designing the experiment and analyzing the data. DX helped with experimental procedures and manuscript preparation. MZ and GZ designed the study and critically revised the manuscript. All authors read and approved the final manuscript.

## References

[B1] Zhang HM Yang J Chen JP Adams MJ A black-streaked dwarf disease on rice in China is caused by a novel fijivirus Arch Virol 2008 153 1893 1898 10.1007/s00705-008-0209-4 18820828

[B2] Zhou GH Wen JJ Cai DJ Li P Xu DL Zhang SG Southern rice black-streaked dwarf virus: A new proposed *Fijivirus* species in the family *Reoviridae* Chinese Sci Bull 2008 53 3677 3685 10.1007/s11434-008-0467-2

[B3] Wang Q Yang J Zhou GH Zhang HM Chen JP Adams MJ The complete genome sequence of two isolates of Southern rice black-streaked dwarf virus, a new member of the Genus *Fijivirus* J Phytopathol 2010 158 733 737 10.1111/j.1439-0434.2010.01679.x

[B4] Pu LL Xie GH Ji CY Ling B Zhang MX Xu DL Zhou G Transmission characteristics of Southern rice black-streaked dwarf virus by rice planthoppers Crop Prot 2012 41 71 76

[B5] Zhou GH Zhang SG Zou SF Xu ZW Zhou ZQ Occurrence and damage analysis of a new rice dwarf disease caused by Southern rice black-streaked dwarf virus Plant Prot 2010 36 144 146 In Chinese

[B6] Guo R Zhou GH Zhang SG Character of Southern rice black-streaked disease and its control strategy China Plant Prot 2010 8 17 20 In Chinese

[B7] Liu Y Jia DS Chen HY Chen Q Xie LH Wu ZJ Wei TY The P7-1 protein of southern rice black-streaked dwarf virus, a fijivirus, induces the formation of tubular structures in insect cells Arch Virol 2011 156 1729 1736 10.1007/s00705-011-1041-9 21671041

[B8] Zhai BP Zhou GH Tao XR Chen X Shen HM Macroscopic patterns and microscopic mechanisms of the outbreak of rice planthoppers and epidemic SRBSDV Chinese J Appl Entomol 2011 48 480 487 In Chinese

[B9] Colvin J Omongo CA Govindappa MR Stevenson PC Maruthi MN Gibson GSS Muniyappa V Host-plant viral infection effects on arthropod-vector population growth, development and behaviour: Management and epidemiological implications Adv Virus Res 2006 67 419 452 17027686 10.1016/S0065-3527(06)67011-5

[B10] Rubinstein G Czosnek H Long-term association of *tomato yellow leaf curl virus* with its whitefly vector *Bemisia tabaci*: Effect on the insect transmission capacity, longevity and fecundity J Gen Virol 1997 78 2683 2689 9349491 10.1099/0022-1317-78-10-2683

[B11] Belliure B Janssen A Maris PC Peters D Sabelis MW Herbivore arthropods benefit from vectoring plant viruses Ecol Lett 2005 8 70 79

[B12] Yoshida S Forno DA Cock JH Gomez KA Laboratory manual for physiological studies of rice, Ed 3 1976 Manila, Philippines: International Rice Research Institute 61 64

[B13] Wang Q Zhou GH Zhang SG Detection of Southern rice black-streaked dwarf virus using one-step dual RT–PCR Acta Phytopathol sin 2012 42 84 87 In Chinese

[B14] Srinivasan R Alvarez JM Bosque-Perez NA Eigenbrode SD Novy RG Effect of an alternate weed host, hairy nightshade, *Solanum sarrachoides*, on the biology of the two most important Potato leafroll virus (Luteoviridae: Polerovirus) vectors, *Myzus persicae* and *Macrosiphum euphorbiae* (Aphididae : Homoptera) Environ Entomol 2008 37 592 600 10.1603/0046-225X(2008)37[592:EOAAWH]2.0.CO;2 18419933

[B15] Jiu M Zhou XP Tong L Xu J Yang X Wan FH Liu SS Vector–virus mutualism accelerates population increase of an invasive whitefly PLoS One 2007 2 e182 10.1371/journal.pone.0000182 17264884 PMC1773017

[B16] Mann RS Sidhu JS Butter NS Sohi AS Sekhon PS Performance of *Bemisia tabaci* (Hemiptera: Aleyrodidae) on healthy and *Cotton Leaf Curl Virus* infected cotton Florida Entomologist 2008 91 249 255 10.1653/0015-4040(2008)91[249:POBTHA]2.0.CO;2

[B17] Sidhu JS Mann RS Butter NS Deleterious effects of *Cotton leaf curl virus* on longevity and fecundity of whitefly, *Bemisia tabaci (Gennadius)* J Entomol 2009 6 62 66 10.3923/je.2009.62.66

[B18] López-Gresa MP Lisón P Kim HK Choi YH Verpoorte R Rodrigo I Conejero V Bellés JM Metabolic fingerprinting of tomato mosaic virus infected *Solanum lycopersicum* J Plant Physiol 2012 169 1586 1596 10.1016/j.jplph.2012.05.021 22795749

[B19] Bosque-Pérez NA Eigenbrode SD The influence of virus-induced changes in plants on aphid vectors: Insights from luteovirus pathosystems Virus Res 2011 159 201 205 10.1016/j.virusres.2011.04.020 21549769

[B20] Jia DS Chen HY Mao QZ Liu Q Wei TY Restriction of viral dissemination from the midgut determines incompetence of small brown planthopper as a vector of Southern rice black-streaked dwarf virus Virus Res 2012 167 404 408 10.1016/j.virusres.2012.05.023 22683297

[B21] Jia DS Chen HY Zheng AL Chen Q Liu Q Xie LH Wu ZJ Wei TY Development of an insect vector cell culture and RNA interference system to investigate the functional role of Fijivirus replication protein J Virol 2012 86 5800 5807 10.1128/JVI.07121-11 22398296 PMC3347266

[B22] Garbutt JS Reynolds SE Induction of RNA interference genes by double-stranded RNA: implications for susceptibility to RNA interference Insect Biochem Mol Biol 2012 42 621 628 10.1016/j.ibmb.2012.05.001 22634162

[B23] Garbutt JS Bellés X Richards EH Reynolds SE Persistence of double-stranded RNA in insect hemolymph as a potential determiner of RNA interference success: Evidence from *Manduca sexta* and *Blattella germanica* J Insect Physiol 2012 http://dx.doi.org/10.1016/j.jinsphys.2012.05.013 10.1016/j.jinsphys.2012.05.013 22664137

[B24] Xu Y Huang LZ Fu S Wu JX Zhou XP Population diversity of *Rice Stripe Virus*-derived siRNAs in three different hosts and RNAi-based anti viral immunity in *Laodelphax striatellus* PLoS One 2012 7 e46238 10.1371/journal.pone.0046238 23029445 PMC3460854

[B25] Zhang L Hou DX Chen X Li DH Zhu LY Zhang YJ Li J Bian Z Liang XY Cai X Yin Y Wang C Zhang TF Zhu DH Zhang DM Xu J Chen Q Ba Y Liu J Wang Q Chen JQ Wang J Wang M Zhang QP Zhang JF Zen K Zhang CY Exogenous plant MIR168a specifically targets mammalian LDLRAP1: evidence of cross-kingdom regulation by microRNA Cell Res 2012 22 107 126 10.1038/cr.2011.158 21931358 PMC3351925

[B26] Xu Y Zhou WW Zhou YJ Wu JX Zhou XP Transcriptome and comparative gene expression analysis of *Sogatella furcifera* (Horváth) in response to Southern Rice Black-Streaked Dwarf Virus PLoS One 2012 7 e36238 10.1371/journal.pone.0036238 22558400 PMC3338671

[B27] Srinivasan R Alvarez JM Effect of mixed viral infections (*Potato virus Y-Potato leaf roll virus*) on biology and preference of vectors *Myzus persicae* and *Macrosiphum euphorbiae* (Hemiptera: Aphididae) J Econ Entomol 2007 100 646 655 10.1603/0022-0493(2007)100[646:EOMVIP]2.0.CO;2 17598521

[B28] Jimenez-Martinez ES Bosque-Perez NA Berger PH Zemetra RS Life history of the bird cherry-oat aphid, *Rhopalosiphum padi* (Homoptera: Aphididae), on transgenic and untransformed wheat challenged with *barley yellow dwarf virus* J Econ Entomol 2004 97 203 212 10.1603/0022-0493-97.2.203 15154437

[B29] Gildow FE Increased production of alates by aphids (Hemiptera, Aphididae) reared on oats infected with *Barley yellow dwarf virus* Ann Entomol Soc Am 1980 73 343 347

[B30] Gildow FE Influence of barley yellow dwarf virus-infected oats and barley on morphology of aphid vectors Phytopathol 1983 73 1196 1199 10.1094/Phyto-73-1196

[B31] D’Amelio R Palermo S Marzachi C Bosco D Influence of Chrysanthemum yellows phytoplasma on the fitness of two of its leafhopper vectors, *Macrosteles quadripunctulatus* and *Euscelidius variegatus* Bull Insectol 2008 61 349 354

[B32] Hogenhout SA Ammar ED Whitfield AE Redinbaugh MG Insect vector interactions with persistently transmitted viruses Annu Rev Phytopathol 2008 146 327 359 18680428 10.1146/annurev.phyto.022508.092135

[B33] Chen SX Wu HL Liao XG The analysis on the prevalent reason of Southern rice black-streaked dwarf virus in the middle of Zhejiang Zhejiang Agric Tech 2000 6 287 289 In Chinese

[B34] Pusag JCA Hemayet Jahan SM Lee KS Lee S Upregulation of temperature susceptibility in *Bemisia tabaci* upon acquisition of *Tomato yellow leaf curl virus* (TYLCV) J Insect Physiol 2012 58 1343 1348 10.1016/j.jinsphys.2012.07.008 22841829

[B35] Stumpf CF Kennedy GG Effects of *tomato spotted wilt virus* isolates, host plants, and temperature on survival, size, and development time of *Frankliniella occidentalis* Entomol Exp Appl 2007 123 139 147 10.1111/j.1570-7458.2007.00541.x

